# Mathematical Geometry and Groups for Low-Symmetry Metal Complex Systems

**DOI:** 10.3390/molecules28114509

**Published:** 2023-06-02

**Authors:** Takashiro Akitsu

**Affiliations:** Department of Chemistry, Faculty of Science, Tokyo University of Science, 1-3 Kagurazaka, Shinjuku-ku, Tokyo 162-8601, Japan; akitsu2@rs.tus.ac.jp; Tel.: +81-3-5228-8271

**Keywords:** geometry, group theory, crystal structure, low symmetry, coordination chemistry

## Abstract

Since chemistry, materials science, and crystallography deal with three-dimensional structures, they use mathematics such as geometry and symmetry. In recent years, the application of topology and mathematics to material design has yielded remarkable results. It can also be seen that differential geometry has been applied to various fields of chemistry for a relatively long time. There is also the possibility of using new mathematics, such as the crystal structure database, which represents big data, for computational chemistry (Hirshfeld surface analysis). On the other hand, group theory (space group and point group) is useful for crystal structures, including determining their electronic properties and the symmetries of molecules with relatively high symmetry. However, these strengths are not exhibited in the low-symmetry molecules that are actually handled. A new use of mathematics for chemical research is required that is suitable for the age when computational chemistry and artificial intelligence can be used.

## 1. Introduction

Mathematical indexes for classifying compounds will be increasingly used in the artificial intelligence era; hence, I would like to provide an overview of the relationship between conventional and present (structural inorganic) chemistry and mathematical geometry or group theory for symmetry treatment. Chemistry (materials science) is a field that elucidates the relationship between the structure and function of substances. The structure of matter has a hierarchy, including its electronic structure, chemical structure, three-dimensional structure, crystal structure, and supramolecular structure, and crystallography still has strengths in structural determination. In recent years, the link between chemistry and mathematics (particularly topology and geometry) has attracted attention from the perspective of introducing geometric descriptors for discussing structures [1]. The mathematicians Kotani et al. proposed a material design method that combines discrete geometric analysis and materials science, for example, non-equilibrium materials with mathematical dynamics, topological functional materials with topology, and multi-scale hierarchical materials with discrete topology [2]. Indeed, they achieved remarkable results in their research on metallic glasses with a focus on crystal symmetry in collaborative works with material scientists [3]. Underlying these developments is thought to be Sunada’s work in the field of topological crystals [4], research that deals mathematically and geometrically with the symmetry of the crystal lattice of diamond [5] and other accumulations. So-called “mathematical crystallography”, as a branch of crystallography, originates from the symmetry of the space group, and the consideration of crystals as mathematical objects is quite different.

In addition to such studies on shape and connection in crystals from the topological viewpoints of compounds [6], Tagami and Kotani et al. also investigated negatively curved cubic carbon crystals with octahedral symmetry [7]. This can easily be judged to be a reliable approach in terms of discussing the three-dimensional structure of crystals and molecules, which are closely related to their electronic structure. In order to describe such a curved surface, topology and differential geometry may play an important role [8,9,10]. In the old literature, we find that differential geometry has been applied to chemistry for three-dimensional structures, and Tachibana and Fukui [11] used this mathematical approach to discuss a potential surface of reactions.

Of course, a three-dimensional structure is the main target for which differential geometry has been used in the field of chemistry. Early on, Louie et al. [12] published work on the use of differential geometry to describe the patterns of protein conformation and dynamics. An important point is the mathematical representation of the analysis of curves and surfaces found in α-helix and β-sheet structures. Naturally, this era was before the rise of bioinformatics; there was still no further development in informatics, and the number of biopolymers whose crystal structures had been analyzed was small. Shortly after this, Blum et al., in discussing the topology of organic compounds, focused on the minimal surface [13] and applied the Bonnet transformation to the discussion of racemization reactions and isomers [14]. In this way, the relationship between structural organic chemistry and differential geometry was born.

Furthermore, having a relationship with electronic structure, Zimple et al. analyzed the relationship between early generalizations of molecular symmetry, called syntrophy, and molecular equivalence based on diffeomorphisms of electron density functional graphs. The Riemannian metric property allows us to choose a suitable reference electron density function for each class of equivalent density. Such reference densities serve in combination with quantum chemical calculations as a tool for the systematic classification of infinite families of electron densities of molecular conformation [15].

The relationship between crystal structure and differential geometry includes its application to descriptions of symmetry operations and modular structures by Kocian et al. [16,17]. The importance of this idea lies in the fact that we regard operations on space groups and point groups as manifold in tangent spaces. Space vectors are probably familiar to crystallographers, but the concept of “manifold” used in geometry and mathematics is not something most crystallographers and chemists (with the exception of a few theoretical physicists) learned at university. It is a concept that has never existed before, and there seems to be a hurdle preventing its widespread adoption.

However, in recent years, with the rise of chemical and materials science research (materials informatics) that fuses with data science, we have begun to see research using differential geometry as a modeling tool. Nguyen et al. [18] reported research using differential geometry to discuss three-dimensional structure data sets, such as the docking of biomolecules and small molecules in drug design and discovery. In such data-driven research, it is expected that the number of research cases where appropriate mathematical tools will improve prospects will increase in the future.

## 2. Symmetry and Structural Inorganic Chemistry

In structural inorganic chemistry, group theory (point groups of molecules and space groups of crystals) has long been used as a form of mathematics to deal with three-dimensional structures. It has been regarded as the “common language’’ of chemists for a long time. Related research methods include recent data science, traditional computational chemistry, and solid-state physics. Originally, crystal structures inherently have a tool called a “space group” to handle symmetry mathematically. The chemical crystallography database (CSD) already contains more than 10,000 records. With the development of computers and programs, not only can crystal structure (or the three-dimensional structure of chemical molecules) data be used as they are, but so can secondary data such as the calculation results of quantum chemistry/electronic structure and intermolecular interactions (e.g., Hirshfeld surface analysis [19]). Hirshfeld surface analysis can visualize information about intermolecular interactions on surfaces surrounding molecules. It is potentially a good partner for information combined with differential geometry. It is also becoming relatively easy to provide and handle relevant information. However, based on the crystal structure of CSD, the argument of minimum symmetry (Banaru et al. [20]), which deals with intermolecular interactions, gives us a better perspective on human mathematics. In this way, “symmetry” will continue to be an important concept no matter how many complicated (low-symmetry) compounds becomes a focus of attention in (inorganic or coordination) chemistry.

A “group” is a kind of set used in mathematics. It is often used in materials science (physics and chemistry) to discuss the symmetry of crystals and molecules as follows: the point group (Koster group), a subset of a broad rotation group (there are 32 crystal point groups); the subperiodic group of low-dimensional translations and rotations (they have 7 bands, 75 bars, and 80 layers); the space group (Fedorov group), with 230 points; the magnetic point group (Shubnikov group), with 122 points; the magnetic subperiodic group, with 31 magnetic band groups, 394 magnetic bar groups, and 528 magnetic layer groups; the magnetic space group, with 1651 points, etc.

In this context, molecules and their attributes are classified by symmetry, and point groups are just one of them. As for point groups and electronic states, ligand field theory (or crystal field theory) is featured in most inorganic chemistry textbooks [21]. The hexa-coordinated octahedral type (*O*_h_), the tetra-coordinated tetrahedral type (*T*_d_), and a slightly derived tetra-coordinated planar type (*D*_4h_) explain how five d orbitals are split by ligand fields. As a typical example, in the hexa-coordinated octahedron (*O*_h_), there are two e_g_ orbitals distributed in six ligand directions under the constraint that the energy level centroids of the five orbitals are conserved. The explanation is that the energy levels are destabilized, while the energy levels of the three t_2g_ orbitals are stabilized, and the splitting width is expressed as 10*D*q. However, the metal complexes that have been studied in recent years do not all have such typical highly symmetrical molecular structures or coordination geometries, or rather few. For this reason, even some difficult students often object to the significance of studying ligand theory. Therefore, in the case of dealing with metal complexes with low symmetry, an example where group theory is not very useful and an example where computational chemistry can be effective will be introduced here.

## 3. Limitations of Group Theory and ‘Actual’ Low-Symmetry Compounds

Most theories have restricted scope and limitations. It is not uncommon not only to deal with compounds that typically have high symmetry but to actually deal with compounds with even lower symmetry. Qualitative molecular orbital methods (ferrocene, etc.) have long been described in textbooks. However, it is no longer realistic to handle the compounds currently being studied using point groups (although the crystal structure is always included in the 230 space groups), and computational chemistry such as DFT is required.

Ferrocene is a good example of a basic metal complex that needs to be handled using the molecular orbital method. It is well known that in the course of the discovery of ferrocene, after it was synthesized, the bonding mode and crystal structure were controversial. Thus, the point group of ferrocene requires caution. The following is a description based on the student’s report in order to identify the points to be noted [22]. Ferrocene has a η^5^-coordination bond in which an iron(II) ion is sandwiched between two cyclopentadienyl ions from above and below, and is called a sandwich complex. Ferrocene is capable of many reactions unique to aromatics and can be synthesized into various derivatives. The best known derivatives are substitutions. The aromaticity of ferrocene is due to its electronic structure. Ordinary ferrocene is coordinated with iron(II) ions, and the upper and lower cyclopentadienyl ions are coordinated in a twisted fashion, which gives a deep yellow (orange) color. On the other hand, each of the two cyclopentadienyl rings has a negative charge of −1. Dispersion transforms the entire cyclopentadienyl ring into a 6π-electron system, which imparts aromaticity to the ring and increases its stability. Additionally, the Fe atom in ferrocene has 18 electrons, including the d^6^ electrons of iron(II), 10 electrons of the cyclopentadienyl ring, and 2 electrons (upper and lower) that are coordinated. That is, it satisfies the 18-electron rule. Therefore, it becomes a more stable complex. In the case of the torsional type, an axis runs from top to bottom, and this is the even-order reflection axis S_10_. Furthermore, the high-order singular axis of rotation C_5_ passes vertically through like S_10_. There are five C_2_ axes running perpendicular to the C_5_ axis. Since the upper and lower rings are twisted, the reflection plane σ_v_ does not exist. However, there are five vertical planes of symmetry that run parallel between the C_2_ axes. Therefore, the staggered forms belong to the D_5d_ group. Similarly, consider lapped ferrocene. Unlike the torsion type, there is no even-order S_n_ axis. There is one C_5_ axis penetrating vertically, and five C_2_ axes passing perpendicularly to the C_5_ axis. A reflection plane σ_v_ exists so as to bisect one angle of the pentagon. Therefore, the overlapping type is the D_5h_ group. Ferrocene exhibits a very stable redox reaction. If cyclopentadienyl is considered a neutral ligand, an iron atom becomes zero-valent. This torsion and stacking are interconvertible via oxidation–reduction.

**Figure 1:**
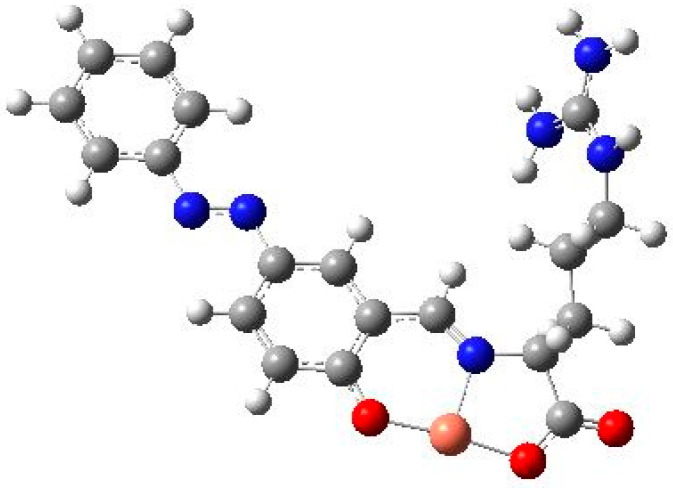
“Tentative model” structure of a *copper(II) complex* with an *L*-amino acid derivative Schiff base ligand (without the forth ligand).

A qualitative molecular orbital method would be the marginal limit of the applicability of the point group. We can proceed further to low-symmetry systems taken from our studies. For example, we studied a copper(II) complex incorporating L-amino acid derivatives, known as Schiff base [23] (Figure 1). Its coordination geometry deviates from the regular square planar one and the tetrahedral one. From the “tentative” result of powder XRD analysis (mistakenly obtained by a graduate student), the space group was labelled P-1, though this chiral molecule (containing an L-amino acid moiety) should not have a center of inversion. However, checking the centrosymmetric structure of the space group is not valid, just like the case of single crystal analysis. If it is isolated as a powder/solid, it forms a crystal and must belong to one of the 230 space groups. Usually, if the space group is as highly symmetrical as possible, the group with high symmetry is included in the group with low symmetry (conversely, the low-symmetry group is a subgroup of the high-symmetry group). In extreme terms, only the lowest symmetric P1 space group is enough for calculations of crystal structures.

As for molecular symmetry, “Molecules without an axis of reflection (rotational axis) S_n_ are chiral.” This is a well-known rule of point groups [24]. The coordination bond angles in cis-positions are between about 85 and 97 degrees, which suggests that it affords a distorted square planar geometry (deviating from regular D_4h_ symmetry). In an experimental study [23], electronic spectra were discussed mainly for the photo-isomerization of the azobenzene moiety of a ligand (focused on π−π* bands), and infrared spectra were used only for the purpose of characterization. Thus, the assignment of a d-d transition based on electronic (or circular dichroism) spectroscopy, as well as vibrational normal modes (if a more complex discussion is needed), should be carried out using the results of computational chemistry, such as density functional theory calculations after structural optimization. This also means that the selection rule for electric dipole transitions and magnetic dipole transitions using quantum mechanical descriptions as products (bra vector (wavefunction of the ground state), X observable (for each spectroscopic analysis), and X ket (wave function of the excited state)) cannot be used. When there is only a P1 space group or only a C_1_ point group, constraints on space group selection due to molecular structure and some useful information related to physical properties (the determination of infrared absorption activity based on chirality and the dipole moment) are lost.

## 4. Conclusions and Future Perspectives

This paper reviews the traditional mathematics of symmetry in terms of structural inorganic chemistry and related aspects. Here, the application limit of the theory is clearly indicated as a problem. In the era of artificial intelligence, data science will likely be used extensively in chemical (materials science) research in the future. Whether or not statistically derived features have physical or chemical meanings or evidence, or whether they can be read by researchers, will be an important bridge to deepening our understanding.

In the meantime, I would like to make an effort to recover “nullified information” by comparing useful arguments based on classical high-symmetry premised group theory with recent arguments based on computational chemistry. The spread of artificial intelligence in the near future will inevitably affect the style of chemical research, in order to scientifically utilize the facts that chemistry research accumulates (or has accumulated). On the contrary, using mathematics as we have done so far may prevent the progression of materials science or chemistry in the future.

## References

[1] Kotani M. (2018). Mathematics and Chemistry-Exploring New Collaborations in a Digital Society. Chem. Chem. Ind..

[2] Kotani M. (2013). A Challenge by Mathematics-Materials Science Collaboration. Hyomen Kagaku.

[3] Hirata A., Kang L.J., Klumov B., Matsue K., Kotani M., Yavari A.R., Chen M.W. (2013). Geometric Frustration of Icosahedron in Metallic Glasses. Science.

[4] Sunada T. (2012). Lecture on topological crystallography. Jpn. J. Math..

[5] Sunada T. (2006). Why do diamonds look so beautiful? Crystal lattices with big symmetry. Expo. Curr. Math..

[6] Flapan E. (2000). When Topology Meets Chemistry: A Topological Look at Molecular Chirality.

[7] Tagami M., Liang Y., Naito H., Kawazoe Y., Kotani M. (2014). Negatively curved cubic carbon crystals with octahedral symmetry. Carbon.

[8] Wadachi M. (1996). Differential & Topological Geometry.

[9] Nagaosa N. (2016). Differential Geometry and Topology.

[10] Nakauchi N. (2011). Geometry Must be Differentiated–Introduction to Differential Geometry.

[11] Tachibana A., Fukui K. (1978). Differential Geometry of Chemically Reacting Systems. Thoret. Chim. Acta.

[12] Louie A.H., Somorjai R.L. (1982). Differential Geometry of Proteins: A Structural and Dynamical Representation of Patterns. J. Theor. Biol..

[13] Blum Z., Lidin S. (1988). The Carcerand, an Organic Structure on a Minimal Surface. Org. Chem. Biochem..

[14] Blum Z., Lidin S., Eberson L. (1988). Differential Geometry and Organic Chemistry; the Bonnet Transformation Applied to the Racemization of Tri-o-thymotide and Isomerization of Cyclophenes. Acta Chem. Scandinavica. Ser. B Org. Chem. Biochem..

[15] Zimpel Z., Mezey P.G. (1997). Molecular Geometry and Symmetry from a Differential Geometry Viewpoint. Int. J. Quantum Chem..

[16] Kocian P., Schenk K., Chapuis G. (2009). Differential geometry: A natural tool for describing symmetry operations. Acta Cryst. A.

[17] Kocian P., Schenk K., Chapuis G. (2010). The role of the tangent bundle for symmetry operations and modulated structures. Acta Cryst. A.

[18] Nguyen D.D., Wei G.-W. (2019). DG-GL: Differential geometry-based geometric learning of molecular datasets. Int. J. Numer. Methods Biomed. Eng..

[19] Spackman M.A., Jayatilaka D. (2009). Hirshfeld surface analysis. CrystEngComm.

[20] Banaru A., Kochnev A. (2017). The minimal set of intermolecular interactions in the structures of substituted prolines. Stud. UBB Chem. LXII.

[21] Yuasa M., Akitsu T. (2014). Fundamental and Applications of Coordination Chemistry.

[22] Akitsu T. (2019). Lecture Note on Inorganic Chemistry.

[23] Kuroda Y., Miyazaki R., Shimonishi D., Nakane D., Akitsu T. (2023). Coordination and Photoisomerization of Azobenzene-Amino Acid Schiff Base Copper(II) Complexes to Lysozyme. J. Mater. Sci. Chem. Eng..

[24] Cotton F.A. (1990). Chemical Applications of Group Theory.

